# Supplementary material for: Learning curve and surgical outcome of robotic assisted colorectal surgery with ERAS program
Source: Sci Rep. 2022 Nov 29;12:20566. doi: 10.1038/s41598-022-24665-w (PMC9709162; doi:10.1038/s41598-022-24665-w)
Supplement: Supplementary file 2 — Supplementary Information 2. [file 41598_2022_24665_MOESM2_ESM.zip › RAL Raw data-Ñ[▒K/085/HS_ñw╝╨░O▒Kñσ.pdf]

正式摘要(電子簽章：000000217599037)	□【讀取】憑證	
W52 / W52 □英文標題(注意:無英文姓名-請補病人基本檔!)		

臺中榮民總醫院

出院病歷摘要

轉入號 : 轉出號 :

[redacted]

**(8)**流水編號	
**(9)**入院日期	110/05/19 (2021) 直腸外科 W52 - 151
**(10)**轉科日期	
**(11)**出院日期	110/05/24 (2021) 住院天數計 5 日

診斷

- 入院 :Rectal cancer
- 出院 :Rectal cancer s/p Robot Assisted Low Anterior Resection + Loop ileostomy on 2021/5/20

主訴

- rectal cancer admitted for Robot Assisted Low Anterior Resection

病史

- The 19-year-old suffered from bloody stool since 2020/10. Endoscopic was done and showed Rectum， 5-8 cm from anal verge， endoscopic biopsy --- Adenocarcinoma， well to moderately differentiated and he was transferred to our hospital. Therefore, under the impression of rectal cancer, he was admitted for Robot Assisted Low Anterior Resection

體檢發現

- PS 70%,
  - Performance status(ECOG)： 0(Normal activity)
  - General appearance :
    - chronic ill looking , conscious clear , without cardiopulmonary distress
    - Vital signs: B.P. 127/58 mmHg , P.R. 113 /min , R.R. 16 /min , B.T. 35.8 ℃
  - HEENT :
    - Head: grossly normal, no deformity
    - Eyes: no anemic conjunctiva, no icteric sclera, normal EOM
    - ENT: no otorrhea, no rhinorrhea, no erythematous mucosa, no injected tonsil, no deformity
  - Neck: supple, freely movable, trachea midline,no deformity, no engorged jugular vein , no palpable thyroid or lymph node enlargement
  - Chest:
    - Inspection: Grossly normal, symmetric and fully expansion, no accessory muscle used, no operation scar, no spider angiomata, no gynecomastia
    - Palpation: no palpable mass or nodule, no subcutaneous emphysema, no tenderness
    - Percussion: resonance throughout bilateral lung field
    - Ausculation: clear breathing sound
  - Heart:
    - Inspection: invisible PMI
    - Palpation: no thrill, no heave
    - Percussion: normal heart size
    - Ausculation: regular heart beat, normal S1, S2, no gallop, no murmur
  - Abdomen:
    - Inspection: in
      - flat contour, symetrically, no scar, no stria, no dilated veins, no rashes

-
  - Ausculation: normoactive bowel sound, no abdominal bruits
  - palpation: soft, no muscle guarding or rigidity, no local tenderness, no rebounding pain, no palpable mass, no palpable pulsation
  - Percussion: no shifting dulllness, no hepatomegaly, no splenomagaly
- Back and spine: no C-V angle knocking pain, no scoliosis, no kyphosis, no lordosis, no deformity
- Extremities: No deformity, full ranged of motion without limitation, no clubbing finger or nail cyanosis, no pitting edema, no ulcer or pigmentation, no obvious skin lesion, no varicose vein, no bone, joint or muscle tenderness
-

Peripheral pulsation :	CA	BA	RA	FA	PA	PTA	DPA
Right side :	++	++	++	++	++	++	++/+++
Left side :	++	++	++	++	++	++	++/+++

- Genito-anus: no tenderness or palpable mass lesion or rectal shelf, normal external genitalia, normal anal tone, brown stool
- Neurological examination normal mental status, alert and cooperative, orientation ok, no sensory or motor impairment, intact cranial nerve function, no pathological reflexes
-

	right upper limbs	left upper limbs	right lower limbs	left lower limbs
Muscle power :	5/5	5/5	5/5	5/5
senosory :	+++	+++	+++	+++

SURGICAL CONDITION

- 1. Imaging studies(Sono , CT of abdomen and chest Films):
  1. .
- 2.
  - Lung
  -

手術日期及方法

- 手術日期及方法20210520 Robot Assisted Low Anterior Resection + Loop ileostomy

---

住院治療經過

- After admission, Rectal cancer s/p Robot Assisted Low Anterior Resection + Loop ileostomy was done on 2021/5/20 under no contraindication of surgery. After surgery, urine retention noted after Foley removal and relieved after catheterization. Under stable condition, he was discharge and arranged OPD followed up.

合併症

一般檢查

- 累積報告 :

DATE	TIME	ALB	TP	BIL.T	BIL.D	ALKP	AST	ALT	LDH	CHOL	TG	HDL-C	LDL	UA	NH3	AMY
1100520	1549	3.6		0.9	0.3	89	10	10								

DATE	TIME	WBC	RBC	HGB	HCT	MCV	PLT	BLST	NEUT	BAND	SEG	LYM	MON	EOS	BAS
1100520	1550	7550	4.37	13.4	40.1	91.8	156		95.9			2.3	1.6	0.1	0.1

DATE	TIME	NA	K	CL	CA	BUN	CREAT	Mg	P	CRP	ESR	PCT	LIPASE	LACTATE
1100520	1549	139	4.3	106	8.1	6	0.97							

DATE	TIME	GLUCOSE	PAB	TRF	ZN	CU	KETONE	CK	CKMB	PT-P	PT-C	APTT-P	APTT-C	TRO-T
1100520	1549	131												

特殊檢查

病理報告

其它

出院時情況

- 3.改門診治療

出院指示

-

藥名	劑量	劑量單位	用法	頻次	數量	數量單位	現況	天數	單位
Ultracet tab	1	TAB	PO	Q6HPRN	12	TAB	TH	3	天
BethanechoL tab 25mg-JS	1	TAB	PO	TIDAC	9	TAB	TH	3	天

出院計劃

主治醫師 2062F 蔣鋒帆 / 住院醫師 9760F 趙英如

正式摘要(電子簽章：**000000217599037)**

W52 / W52

doctorRank:B
